# Supplementary material for: Current Status of the Self-Expandable Metal Stent as a Bridge to Surgery Versus Emergency Surgery in Colorectal Cancer: Results from an Updated Systematic Review and Meta-Analysis of the Literature
Source: Medicina (Kaunas). 2021 Mar 15;57(3):268. doi: 10.3390/medicina57030268 (PMC7998540; doi:10.3390/medicina57030268)
Supplement: Supplementary file 1 [file medicina-57-00268-s001.pdf]

Supplement 1.

Author	Mean age		Sex M: F		Stage IV	
	SEMS group	Surgery group	SEMS group	Surgery group	SEMS group	Surgery group
Cheung et al., 2009	59	62.5	14:10	12:12	2	11
Alcántara et al., 2011	71.9	71.15	5:10	7:6	2	2
Cui et al., 2011	N.A.	N.A.	N.A.	N.A.	N.A.	N.A.
Van Hooft et al., 2011	70.4	71.4	24:23	27:24	N.A.	N.A.
Pirlet et al., 2011	70.4	74.7	16:14	13:17	0	0
Ho et al., 2012	68	65.75	13:7	9:10	3	7
Ghazal et al., 2013	52.25	51	12:18	19:11	0	0
Arezzo et al., 2017	66.5	64	28:28	32:27	0	0
Elwan et al., 2020	59.6	60.3	18:12	20:10	N.A.	N.A.
Arezzo et al., 2020	72 (range 43-98)	71 (range 44-94)	28:28	32:27	3	4

Supplement 2.

<table>
<tr><th rowspan="2">Author</th><th colspan="8">Colorectal cancer location</th></tr>
<tr><th>Right colon</th><th>Transverse colon</th><th>Splenic flexure</th><th>Descending colon</th><th>Sigmoid colon</th><th>Rectosigmoid</th><th>Rectum</th><th>Not re-ported</th></tr>
<tr><td>Cheung et al., 2009</td><td>0</td><td>0</td><td>0</td><td>0</td><td>0</td><td>0</td><td>0</td><td>48</td></tr>
<tr><td>Alcántara et al., 2011</td><td>0</td><td>0</td><td>6</td><td>3</td><td>15</td><td>3</td><td>1</td><td>0</td></tr>
<tr><td>Cui et al., 2011</td><td>0</td><td>0</td><td>0</td><td>49</td><td>0</td><td>0</td><td>0</td><td>0</td></tr>
<tr><td>Van Hooft et al., 2011</td><td>0</td><td>0</td><td colspan="4">98</td><td>0</td><td>0</td></tr>
<tr><td>Pirlet et al., 2011</td><td>0</td><td>0</td><td>3</td><td>8</td><td>33</td><td>15</td><td>0</td><td>1</td></tr>
<tr><td>Ho et al., 2012</td><td>0</td><td>0</td><td>4</td><td>9</td><td>18</td><td>8</td><td>0</td><td>0</td></tr>
<tr><td>Ghazal et al., 2013</td><td>0</td><td>0</td><td>0</td><td>7</td><td>31</td><td>22</td><td>0</td><td>0</td></tr>
<tr><td>Arezzo et al., 2017</td><td>0</td><td>0</td><td>18</td><td>77</td><td>20</td><td>0</td><td>0</td><td>0</td></tr>
<tr><td>Elwan et al., 2020</td><td>9</td><td>0</td><td>0</td><td>51</td><td>0</td><td>0</td><td>0</td><td>0</td></tr>
<tr><td>Arezzo et al.,2020</td><td>0</td><td>0</td><td>18</td><td>77</td><td>20</td><td>0</td><td>0</td><td>0</td></tr>
</table>

Supplement 3.

Author	Type of surgical access for colonic resection		Emergency surgery	
	SEMS group	Surgery group	Type surgery	Intraoperative colonic lavage
Cheung et al., 2009	Lap	Open	TACIR, HP, RPA	Yes
Alcántara et al., 2011	Open	Open	HP, RPA	Yes
Cui et al., 2011	Lap	Open	NR	NR
Van Hooft et al., 2011	Open/Lap	Open/Lap	HP, RPA, DC	NR
Pirlet et al., 2011	Lap	Open	TACIR, HP, RPA, DC	NR
Ho et al., 2012	Open/Lap	Open	TACIR, HP, RPA, DC	NR
Ghazal et al., 2013	Open	Open	TACIR	NR
Arezzo et al., 2017	Open/Lap	Open/Lap	TACIR, HP, RPA,	NR
Elwan et al., 2020	NR	NR	TACIR, HP, RPA, DC	Yes
Arezzo et al.,2020	Open/Lap	Open	HP,TACIR,RPA,DC	Yes

Lap: Laparoscopy

TACIR: Total or Subtotal Abdominal Colectomy and Ileorectal Anastomosis

HP: Hartmann's Procedure

RPA: Resection and Primary Anastomosis

DC: Derivative Colostomy

Supplement 4.

Author	Stent type	The time between stent placement and elective surgery (days)	Perforation rate
Cheung et al., 2009	Wallstent	median of 10 days (2-16 days)	0
Alcántara et al., 2011	Wallflex	5–7 days	NR
Cui et al., 2011	N.R.	median of 3,10 days	NR
Van Hooft et al., 2011	Wallstent or Wallflex	5-14 days	12%
Pirlet et al., 2011	Bard Nitinol uncovered self-expanding stent	7 days (5–19 days)	14%
Ho et al., 2012	Wallflex	10 days (9–38 days)	0
Ghazal et al., 2013	Wallflex	7–10 days	0
Arezzo et al., 2017	WallFlex/ Hanaro	10 days (3–8 days)	8.92%
Elwan et al., 2020	Wallflex	NR	0
Arezzo et al.,2020	WallFlex/ Hanaro	3-8 days	8.9%

Supplement 5a.

Risk of Bias graph: review authors' judgements regarding the risk of bias for each item presented as percentages for all included studies.

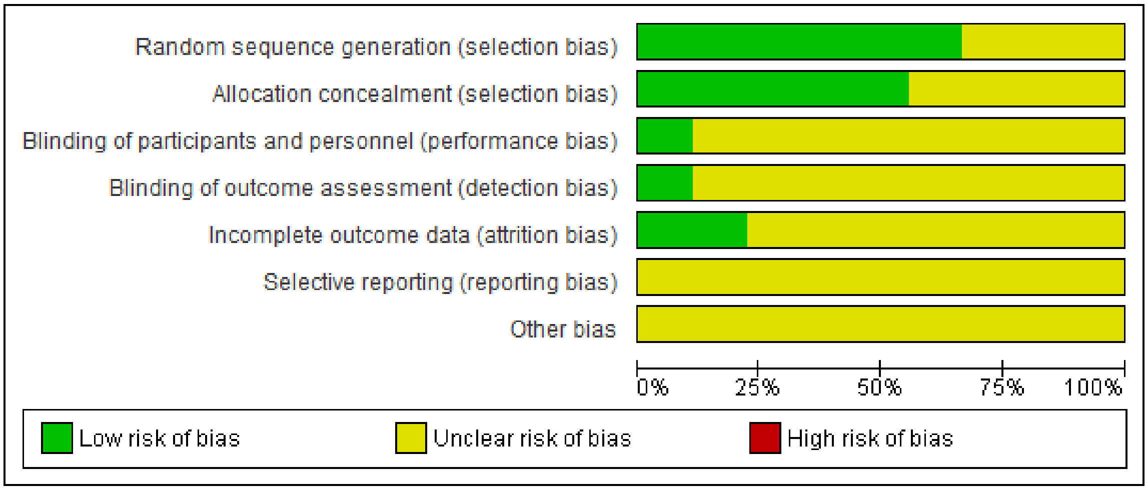

Supplement 5b.

Risk of Bias Summary: review authors' judgements regarding the risk of bias for each of the included studies.

	Random sequence generation (selection bias)	Allocation concealment (selection bias)	Blinding of participants and personnel (performance bias)	Blinding of outcome assessment (detection bias)	Incomplete outcome data (attrition bias)	Selective reporting (reporting bias)	Other bias
Alcantara 2011	?	?	?	?	?	?	?
Arezzo 2016	+	+	?	?	?	?	?
Cheung 2009	+	?	?	?	?	?	?
Cui 2011	?	?	?	?	?	?	?
Elwan 2020	?	?	?	?	+	?	?
Ghazal 2013	+	+	?	?	?	?	?
Ho 2012	+	+	?	?	?	?	?
Pirlet 2011	+	+	?	?	?	?	?
van Hooft 2011	+	+	+	+	+	?	?
